# Bartonella quintana Endocarditis Complicated by Cerebral Stroke: A Case Report

**DOI:** 10.7759/cureus.76074

**Published:** 2024-12-20

**Authors:** Maria Angeles Argudín, Samy Mzougui, Frédéric Frippiat, Jean-Baptiste Giot, Vincent Infantino, Cécile Meex, Marie-Pierre Hayette, Sebastien Bontems, Giulia Zorzi, Anaïs Scohy, Hector Rodriguez-Villalobos, Benoît Kabamba Mukadi

**Affiliations:** 1 Microbiology, Cliniques Universitaires Saint Luc - Université Catholique de Louvain, Brussels, BEL; 2 Medical Microbiology, Institute of Experimental and Clinical Research - Université Catholique de Louvain, Brussels, BEL; 3 National Reference Center Bartonella, Cliniques Universitaires Saint Luc - Université Catholique de Louvain, Brussels, BEL; 4 Clinical Microbiology, University Hospital of Liège, Liège, BEL; 5 Infectiology, University Hospital of Liège, Liège, BEL

**Keywords:** 16srrna, bartonella, csf, encephalitis, endocarditis, mitral valve, qpcr, refugee, serology

## Abstract

*Bartonella quintana* is rarely associated with neurological manifestations. This report describes a rare case of endocarditis complicated by a cerebral stroke caused by *B. quintana*. We also briefly reviewed the neurological clinical spectrum of *B. quintana* disease described in the literature.

Serology tests were performed using the VIRCLIA®-system (chemiluminescence assay, Vircell, Spain) and immunofluorescence assay (IFA; Focus Diagnostics, USA). Cerebrospinal fluid (CSF) was tested using the BioFire-CSF-FilmArray-Meningitis/Encephalitis Panel (bioMérieux, France). CSF, plasma, and biopsy samples were tested using *Bartonella*-qPCR combined with Sanger-sequencing.

A 23-year-old male Afghan refugee residing in Belgium presented with persistent fatigue and cough. A calcified aortic bicuspid with severe insufficiency and moderately associated stenosis was diagnosed. A transesophageal echocardiogram revealed a shrinking valve and a mobile mass attached to the calcification of the free edge at the aorta. He developed fever, a moderate inflammatory syndrome with normocytic anemia, and renal failure with hematuria and proteinuria, indicating probable glomerulonephritis. He met the Duke criteria for infective endocarditis, though blood cultures were initially negative. *Bartonella* serology later returned positive. He developed a fever and intense headache. CSF showed moderate pleiocytosis, but a negative-FilmArray. Neurovascular MR-angiography revealed a multifocal ischemic stroke. His aortic valve was replaced (Ross procedure). The biopsy showed nodular and degenerative fibro-calcified rearrangements.* B. quintana* presence was confirmed in CSF, blood, and mitral valve samples.

Our report underlines that *B. quintana* is a rare but a possible cause of endocarditis and neurological damage, and emphasizes the need for effective healthcare access, which is often limited in the countries of origin of migrants and even for migrants residing in high-resource countries.

## Introduction

*Bartonella quintana* is the causal agent of trench fever. Infection in immunocompetent hosts may result in febrile illness or afebrile bacteraemia with nonspecific symptoms such as headache, conjunctivitis, maculopapular rash, organomegaly, arthralgia, myalgia, and bone pains [1]. The infection is globally distributed but remains endemic in low-income regions, particularly in some African and Asian countries [2]. Outbreaks are associated with poverty, overcrowding, poor sanitation and hygiene [2, 3]. The body louse *Pediculus humanus* is the main vector, though it has also been linked to head lice and cat scratches [2-4]. The spectrum of *B. quintana*-associated disease has expanded in recent years to include angiomatosis in immunocompromised hosts and endocarditis. Additionally, it has been rarely associated with encephalitis [1, 5-11].

Serology tests were performed using the VIRCLIA® automated system (chemiluminescence assay, Vircell, Spain), and *Bartonella *titers were obtained through immunofluorescence assay (IFA; Focus Diagnostics, USA). Cerebrospinal fluid (CSF) samples were tested with the BioFire CSF FilmArray Meningitis/Encephalitis Panel (bioMérieux, France) and a homemade Herpes Virus real-time PCR (qPCR) [12]. Biopsy, plasma, and CSF samples were analyzed with a 16rRNA NGS technique [13], and a *Bartonella *qPCR followed by Sanger sequencing [14]. Prior to DNA extraction, the biopsy sample underwent complete tissue lysis through incubation in 180 μL ATL buffer (QIAGEN, Netherlands) and 20 μL proteinase K (QIAGEN, Netherlands) at 65°C for 30 minutes with shaking at 900 rpm, followed by an additional incubation at 95°C for 10 minutes. Bacterial DNA was extracted using the ELITe InGenius® SP 1000 kit on the ELITe InGenius® instrument (ELITechGroup, France).

This article was previously presented as a meeting abstract and a poster at the 2024 Symposium on Diagnostic and Surveillance of Infectious Diseases (Sciensano) on May 16, 2024.

## Case presentation

A 23-year-old male refugee from Afghanistan, residing at the Red Cross reception centre for asylum seekers in Fraipont (Belgium) since 2021, was referred to the cardiology department of the University Hospital of Liege, Belgium, in late June 2023. Due to a language barrier, anamnesis was limited. The patient presented with a cough, night sweats, dyspnea and a mitral murmur (vitals were normal). He had no prior medical-surgical history. A transthoracic echocardiogram revealed a calcified aortic bicuspid with severe insufficiency (stage 3 out of 4) and moderate associated stenosis (day 1). Cardiac surgery (Ross operation) was scheduled.

**Figure 1:**
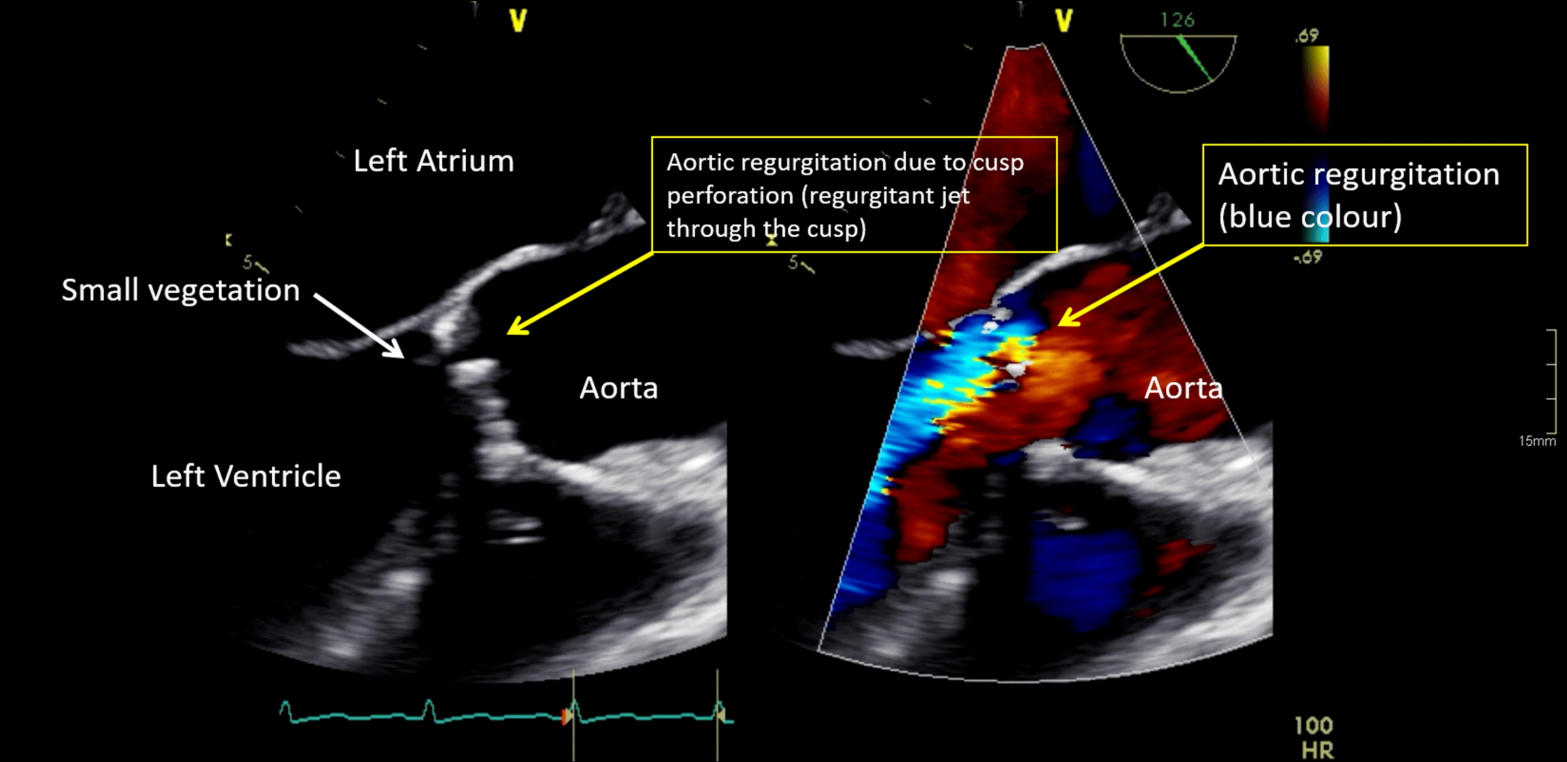
Transesophageal echocardiogram performed on day 11.

Ten days later, he visited the emergency department complaining of persistent cough and fatigue lasting several days (vitals were normal). He did not present with dyspnea, orthopnea, chest pain or fever. A transesophageal echocardiogram revealed a severely shrinking valve and a mobile mass measuring 0.4x0.9 cm attached to the calcification of the free edge at the aorta (Figure 1). The next day, he developed a fever, and an inflammatory syndrome with a normocytic anemia, leading to his hospitalization in the cardiology department for valvular heart disease (vitals were normal). No Osler's nodules were observed on clinical examination. The patient showed normally colored and well-hydrated skin, with no other notable observations. Additionally, renal failure of unknown duration was detected, accompanied by hematuria and proteinuria, suggesting a probable glomerulonephritis potentially related to infective endocarditis. Rheumatoid factor was negative. Thoracic X-ray (days 11 and 15) and a chest computed tomography (CT) scan (day 19) showed no infectious foci. Although, vascular overload was suspected due to the clinical context. A fundoscopic exam (day 17) revealed Roth's and cotton wool spots in both eyes. A positron emission tomography (PET) CT scan (day 23), showed no pathological hypermetabolism next to the aortic valve and no highlighted infectious lesions. Based on one major and three minor Duke criteria, a diagnosis of definite endocarditis was highly suspected.

Repeated sets of blood cultures (from days 17 to 30) were negative. Serological tests on day 19 were negative for *Brucella*, but positive for *Coxiella *and *Bartonella*. The *Coxiella *serology was positive only for phase II IgM and interpreted as nonspecific anti-*Coxiella *IgM, as a control performed on day 36 turned negative. However, *Bartonella *serology was strongly positive on day 19 (Virclia index at 4.5, and titer 1:8192) and day 31 (Virclia index at 3.7 and titer 1:4096). A *Bartonella *qPCR was requested on a plasma sample drawn on day 23.

On day 26, the patient developed a fever and headache. CSF examination revealed an inflammatory cytology with 253 nucleated cells, 81% of which were neutrophils. The BioFire CSF FilmArray Meningitis/Encephalitis Panel (bioMérieux, France) and a homemade Herpes Virus qPCR were negative. Empiric antibiotic therapy, consisting of intravenous ceftriaxone with amoxicillin, was initiated the same day. A neurovascular MR angiography (MRA) performed on day 30 to investigate potential septic embolism, revealed multifocal ischemic strokes, consisting of subacute fronto-parieto temporal and left claustrum, without hemorrhagic transformation or vascular obstruction. Two hemorrhagic formations with discreet contrast were also identified within the right parietal and occipital folds corresponding to probable venous-type vascular malformations. The patient has no other physical or neurological findings apart from headaches. The next day, a transesophageal echocardiogram was performed, showing findings consistent with those from day 11. Given the suspicion of cerebral septic embolism, a lumbar puncture was conducted the same day. But cytological examination of the CSF sample was negative. On days 30 and 31, respectively, doxycycline and gentamicin were added to the treatment for blood culture-negative endocarditis. The fever subsided within a few days. A *Bartonella *qPCR was requested on the CSF sample obtained on day 31. A blood sample drawn on day 32 yielded a negative result by 16SrRNA NGS on day 36.

The aortic valve was replaced via the Ross procedure on day 40. The ascending aorta was replaced via femoro-femoral bypass for aortic insufficiency due to bicuspid and aortic endocarditis. No postoperative complications were observed. The aortic valve biopsy showed nodular and degenerative fibro-calcified rearrangements. No organisms were visualized on Gram staining. 16SRNA NGS and *Bartonella *qPCR were requested on the mitral valve.

On day 47, amoxicillin and ceftriaxone were discontinued following a low positive PCR result for *B. quintana* on the CSF sample from day 31 (Cq 35). On days 51 and 53 respectively, *B. quintana* was also detected on the blood sample from day 23 (Cq 34) and on the mitral valve (Cq 26), confirming the diagnosis of *Bartonella *endocarditis. Gentamicin was discontinued on day 54 due to the patient showing signs of acute kidney injury, likely related to aminoglycoside toxicity. To facilitate discharge, an oral regimen was prioritized. Dual therapy remained a key objective, with rifampin identified as the preferred option, particularly in the context of valve replacement. However, known drug interactions between doxycycline and rifampin required an adjustment [15]. As a result, doxycycline was replaced with minocycline, an alternative tetracycline without such interactions. Therefore, doxycycline was switched to a combination of minocycline and rifampicin on day 57. This treatment was conducted in an outpatient setting and was discontinued on day 99 during a follow-up consultation, as the patient had fully recovered. Evidence of clinical recovery includes the resolution of the inflammatory syndrome, demonstrated by normalized neutrophil levels (4,120/mm³, 63.8%) from day 57 and a negative CRP result (2.0 mg/L) from day 93.

## Discussion

A variety of *Bartonella *species have been documented in conditions affecting both the peripheral and central nervous systems [16]. Neuroretinitis, encephalitis, and encephalopathy are most commonly associated with *B. henselae*. However, similar conditions have also been reported in infections with *B. quintana*, *B. washoensis* (from ground squirrels), and *B. vinsonii* subsp. *vinsonii *(from voles) [16]. Neurological manifestations due to *B. quintana* have been rarely reported (Table 1). Half of the cases are related to neuroretinitis, but *B. quintana *can also cause various forms of intraocular inflammations including Parinaud’s oculoglandular syndrome, retinitis, vasculitis, anterior, intermediate and posterior uveitis [17]. The two encephalopathy cases and the one meningoencephalitis case were reported in immunocompetent hosts. Only one previous case of neurological manifestations following endocarditis caused by *B. quintana* has been reported [11]. This patient, like ours, was a refugee residing in an asylum seekers’ centre in Germany and recalled a massive body louse infestation in a refugee camp [11].

**Table 1:** Reported neurological manifestations by B. quintana. BQ, *B. quintana*; BH, *B. henselae*; EIA, enzyme immunoassay; IFA, indirect immunofluorescence assay; FISH, Fluorescent In-Situ Hybridization.

Case	Patient Age and Sex	Country	Pre-existing conditions	Exposure to possible vectors	Neurological manifestation	B. quintana diagnostic	Reference
1	19-year-old male	United States of America	Epilepsy; chronic otitis media leading to hearing loss; previous interventions/hospitalizations for fibromatosis on the left palm, a perirectal abscess, tonsillitis and pneumonia	Not determined	Granulomatous process involving the right thalamus and surrounding tissues	PCR and sequencing (BH-IgG by EIA serology negative)	[1]
2	8-year-old male	United States of America	Medical history unremarkable	Cat scratch	Encephalopathy without evidence of focal involvement pain	PCR and sequencing (BH-IgG by EIA serology positive)	[1]
3	30-year-old male	United States of America	“Living as a transient”, intravenous drug user	Intravenous drug user	Neuroretinitis	IFA serology (BQ-IgG positive; BH-IgG strongly positive)	[5]
4	16-month-old female	Greece	Medical history was unremarkable	Not determined	Encephalopathy complicated by Guillain-Barre syndrome and hydrocephalus	IFA serology (BQ-IgG positive; BH-IgG positive) + PCR and sequencing	[6]
5	57-year-old female	Croatia	Medical history was unremarkable	Living in a rural setting	Neuroretinitis	IFA serology (BQ-IgG positive; BH-IgG borderline)	[7]
6	20-year-old female	Tunisia	Medical history was unremarkable	Living in a rural setting, contact with animals	Meningoencephalitis	IFA serology (BQ-IgG positive)	[8]
7	12-year-old female	Brazil	Medical history was unremarkable	Contact with animals including cats	Neuroretinitis with stellate maculopathy	IFA serology (BQ-IgG positive; BH-IgG positive)	[9]
8	60-year-old female	United States of America	Hypertension, hypothyroidism	Contact with cat	Neuroretinitis	Antibodies with reflex titers (BQ-IgG positive)	[10]
9	24-year-old female	Germany	Refugee status, cardiomyopathy, slightly reduced left ventricular ejection fraction, aortic valve insufficiency Grade II, bacteremia, several intracerebral hemorrhage events	Previous infestation of body lice in the refugee camps in Libya	Multi-stage intracerebral septic embolism with secondary bleeding (+ infectious aortic and mitral valve endocarditis)	FISH	[11]

In general, migrants do not pose a healthcare threat to the host population. However, specific subgroups, such as refugees, asylum seekers, and irregular migrants, are particularly vulnerable to infectious diseases [18, 19]. Their heightened risk of certain infections stems primarily from poor living conditions during and after migration. Refugee camps and temporary shelters are often overcrowded, with limited access to adequate sanitation and healthcare facilities [18, 19]. These conditions create an environment that facilitates the transmission of infectious diseases, including those vectored by human lice [2, 3]. The body louse *Pediculus humanus* is the main vector, but* B. quintana* DNA has been detected in human head lice and other arthropods, including cat fleas, pigeon mites, bedbugs, and ticks of various species [2, 3]. Impoverished and overcrowded conditions, poor hygiene, a turbulent environment, and body louse infections are the primary risk factors for *B. quintana* infections [3]. Outbreaks also occurred among homeless populations and indigenous communities [2].

Additionally, imported infectious diseases in migrants can present diagnostic challenges for physicians in receiving countries due to limited familiarity with these conditions. Examples are tuberculosis, malaria and vaccine-preventable diseases [18, 19]. Furthermore, migrants may encounter various barriers to accessing healthcare and essential medicines, including systemic restrictions, administrative and financial obstacles, and language barriers. As a result, despite the current effort in European countries to ensure equal access to healthcare for this vulnerable population, disparities in healthcare access between migrants and non-migrants persist [20, 21]. For example, in our case, the patient sought healthcare in an advanced state of illness. Initially, the anamnesis was limited due to a language barrier, which was later improved with the help of an interpreter. Indeed, language barriers can significantly influence access, communication and understanding in healthcare settings [22].

Independent of migrant status, racial and socioeconomic factors also contribute to disease development. While cardiovascular diseases remain the leading cause of death worldwide, lower socioeconomic status is also associated with an increased risk of cardiovascular conditions [23].

The challenges in diagnosis are often and perhaps more importantly, related to the technical difficulties in identifying the pathogen, which necessitate the use of advanced techniques. The diagnostic methods for detecting *B. quintana* include blood culture, serological tests, PCR, and IFA [3]. However, culturing *B. quintana* from blood is challenging, and serological assays can exhibit cross-reactivity with *B. henselae* and *C. burnetii* [3]. *B. quintana* can be the cause of culture-negative endocarditis, and its detection typically requires specific molecular techniques, including PCR and/or sequencing, for species-level identification [2]. Without access to these advanced methods, the exclusion of infective endocarditis may lead to differential diagnoses, such as nonbacterial thrombotic endocarditis, also known as marantic endocarditis [24].

## Conclusions

Neurobartonelloses are primarily associated with *B. henselae* infections, but the number of cases linked to *B. quintana* is rising. Our report highlights that *B. quintana *is a rare but possible cause of culture-negative endocarditis with associated neurological damage. Infections are endemic in low-income regions, but outbreaks linked to poverty, overcrowding, and poor sanitation and hygiene also occur in medium- and high-income countries. Migrant populations, the homeless, and indigenous communities are the most vulnerable. This case report also underscores the need for and benefits of effective healthcare access. However, it is unfortunately still too often unavailable in migrants’ countries of origin, and sometimes even for migrants residing in high-resource countries.
